# Efficacy of the novel heart attack centre extension pathway: a pilot study

**DOI:** 10.1186/cc10787

**Published:** 2012-03-20

**Authors:** D Perera, B Hoonjan, K Krishnathasan, M Selvanyagam, H Neugebauer

**Affiliations:** 1 Barts and The London School of Medicine and Dentistry, London, UK; 2 Queens Hospital, Barking Havering and Redbridge NHS Trust, London, UK

## Introduction

The Barts and the London Heart Attack Centre Extension (HACX) programme was introduced to provide a direct pathway for high-risk non-ST elevation myocardial infarction (NSTEMI) patients from the A&E of a district general hospital to a tertiary intervention centre. As a result, patients have earlier access to angiography and subsequent treatment, including percutaneous coronary intervention (PCI), coronary artery bypass grafting (CABG) or nonsurgical interventions. There is no research on the effectiveness of this novel HACX programme.

### Methods

Over 3 months, 33 patients transferred via the HACX pathway and 37 patients transferred via the conventional interhospital transfer pathway (IHT) were followed up. All patients with acute coronary syndrome symptoms, relevant ECG changes (ST segment depression in two or more contiguous leads >1 mm, pathological T-wave inversion in V1 to V4, a GRACE score >88 and troponin I levels >0.1 ng/ml) were discussed with the cardiology team at the interventional centre prior to immediate transfer. We assessed patient suitability for angiography, post-angiography procedures, and 3-month mortality outcomes. Data were obtained from the hospital's PAS computer system.

## Results

The average time for patients to have an angiography via the IHT pathway was 5.5 days. Of the 33 patients (mean age 61 ± 15.2 SD) transferred via HACX, 30 patients (91%) were appropriately identified for an angiogram. Seventeen patients (52%) required PCI, five patients (15%) required CABG, four patients (12%) required nonsurgical intervention, and four patients (12%) required no treatment. Controls included 37 patients (mean age 71 ± 12.6 SD) of whom 17 patients (46%) required PCI, six patients (16%) required CABG, eight patients (22%) required nonsurgical intervention and six patients (16%) required no treatment. At 3-month follow-up, 32 patients (97%) in the HACX cohort and 36 patients (97%) in the IHT cohort were alive.

## Conclusion

HACX is an effective pathway that accurately identifies and rapidly transfers appropriate NSTEMI patients requiring early coronary revascularisation. However, there was no additional mortality benefit at 3-month follow-up compared to the conventional IHT pathway. Further studies with larger patient cohorts and longer follow-up periods are required to substantiate the benefits of the HACX programme in order to consider whether this service could be implemented nationwide, or whether this is a service that does not need to exist at all.

